# Visfatin induces ovarian cancer resistance to anoikis by regulating mitochondrial activity

**DOI:** 10.1007/s12020-023-03305-x

**Published:** 2023-01-19

**Authors:** Justyna Gogola-Mruk, Wacław Tworzydło, Kinga Krawczyk, Weronika Marynowicz, Anna Ptak

**Affiliations:** 1 Laboratory of Physiology and Toxicology of Reproduction, Institute of Zoology and Biomedical Research, Jagiellonian University, Krakow, Poland; 2 Department of Developmental Biology and Invertebrate Morphology, Institute of Zoology and Biomedical Research, Jagiellonian University, Krakow, Poland

**Keywords:** Visfatin, Anoikis, FK866, Mitochondrial activity, Ovarian cancer

## Abstract

**Purpose:**

Ovarian cancer is characterized by recurrent peritoneal and distant metastasis. To survive in a non-adherent state, floating ovarian cancer spheroids develop mechanisms to resist anoikis. Moreover, ascitic fluid from ovarian cancer patients contains high levels of visfatin with anti-apoptotic properties. However, the mechanism by which visfatin induces anoikis resistance in ovarian cancer spheroids remains unknown. Here, we aimed to assess wheather visfatin which possess anti-apoptotic properties can induce resistance of anoikis in ovarian cancer spheroids.

**Methods:**

Visfatin synthesis were examined using a commercial human visfatin ELISA Kit. Spheroid were exposed to visfatin and cell viability and caspase *3/7 activity* were measured using CellTiter-Glo 3D cell viability assay and Caspase-Glo® 3/7 Assay System. mRNA and protein expression were analyzed by Real-time PCR and Western Blot analysis, respectively. Analysis of mitochondrial activity was estimated by JC-1 staining.

**Results:**

First, our results suggested higher expression and secretion of visfatin by epithelial than by granulosa ovarian cells, and in non-cancer tissues versus cancer tissues. Interestingly, visfatin increased the proliferation/apoptosis ratio in ovarian cancer spheroids. Specifically, both the intrinsic and extrinsic pathways of anoikis were regulated by visfatin. Moreover, the effect of the visfatin inhibitor (FK866) was opposite to that of visfatin. Furthermore, both NAMPT and FK866 affected mitochondrial activity in ovarian cancer cells.

**Conclusion:**

In conclusion, visfatin acts as an anti-apoptotic factor by regulating mitochondrial activity, leading to anoikis resistance in ovarian cancer spheroids. The finding suggest visfatin as a potential novel therapeutic target for the treatment of ovarian carcinoma with peritoneal dissemination.

## Introduction

Ovarian cancer, the gynecological malignancy with the highest mortality rate, is characterized by recurrent peritoneal and distant metastasis. Clinical and case studies report that ascitic fluid contains ovarian cancer cells, either as single cells or as spheroid-like structures, which drive peritoneal dissemination [1, 2]. As a barrier against metastases, normal cells undergo apoptosis after they lose contact with the extracellular matrix or neighboring cells. This cell death process is called “anoikis” [3]. Floating ovarian cancer spheroids have developed mechanisms to resist anoikis, and so have the ability to survive in a non-adherent state while traveling through the circulatory and lymphatic systems [4, 5]. Anoikis involves two apoptotic pathways: the cell death receptor (extrinsic) and mitochondrial (intrinsic) pathways. Likewise, blockade of the mitochondrial pathway, for example, via over-expression of anti-apoptotic Bcl-2 family proteins, can also induce resistance to anoikis [6].

Interestingly, ascites from ovarian cancer patients contains significantly higher levels of visfatin (75.3 ± 28.1 ng/mL) than serum; these high levels are associated intraperitoneal dissemination of ovarian cancer. The source of visfatin in ascites fluid may be both ascites-derived ovarian cancer cells and/or peritoneal and omental adipocytes, which release soluble factors into the ascites and provide a pro-tumor microenvironment [7]. Visfatin is an adipocytokine and cytosolic enzyme with nicotinamide phosphoribosyltransferase (Nampt) activity in mammals. Furthermore, studies show that visfatin inhibits apoptosis of endometrial cancer [8] and breast cancer [9] cells. However, it is unclear whether visfatin triggers anoikis resistance in ovarian cancer spheroids.

The above data raise the intriguing hypothesis that visfatin present in ascites fluid of ovarian cancer patients possess anti-apoptotic properties that drive anoikis resistance in ovarian cancer spheroids. Therefore, the main aim of this study was to examine involvement of visfatin in triggering anoikis resistance in ovarian cancer spheroids, taking into account the special role of mitochondria in anoikis.

## Materials and methods

### Cell culture and chemicals

Three human ovarian cancer cell lines derived from ascitic fluid or the peritoneum were used as an in vitro model to investigate ovarian cancer cell tumorigenesis. These were the human epithelial ovarian carcinoma cell line OVCAR-3 (ATCC, Manassas, VA, USA); the human epithelial ovarian cancer cell line SKOV-3 (ATCC, Manassas, VI, USA); and the human AGCT-derived cell line KGN (Riken Cell Bank (RBRC-RCB1154), Ibaraki, Japan; approved by Drs. Yoshiro Nishi and Toshihiko Yanase). The human ovarian surface epithelial cell line HOSEpiC (ScienCell Research Laboratories, Carlsbad, CA), and a human non-luteinized granulosa cell line derived from antral follicles (HGrC1; a kind gift from Dr. Ikara Iwase (Nagoya University, Japan)) were used as non-cancer controls. OVCAR-3 cells were cultured in RPMI 1640 medium without phenol red, supplemented with 10% heat-inactivated, charcoal-stripped fetal bovine serum (FBS) (Biowest, Nuaillé, France). KGN cells were cultured in DMEM/Ham’s F12 medium without phenol red (Thermo Fisher Scientific), supplemented with 10% FBS. SKOV-3 cells were maintained in McCoy’s 5A medium (Sigma-Aldrich, St. Louis, MO, USA);) supplemented with 10% FBS. HOSEpiC cells were cultured in phenol red-free RPMI 1640 (Thermo Fisher Scientific) supplemented with 10% FBS. HGrC1 cells were cultured in phenol red-free DMEM (Sigma-Aldrich, St. Louis, MO, USA) supplemented with 2 mM L-glutamine and 10% FBS. All cells were maintained in a humidified incubator under 5% CO_2_ at 37 °C. Recombinant human visfatin (NAMPT) (R&D Systems, Inc., Minneapolis, MN, USA) was dissolved in PBS (Thermo Fisher Scientific, Waltham, MA, USA). A potent visfatin inhibitor (FK866; R&D Systems, Inc.,) was dissolved in dimethyl sulfoxide (DMSO). The final concentration of DMSO in the media was <0.1% (v/v).

### CSIOVDB: a microarray gene expression database for ovarian cancer subtype analysis

CSIOVDB, a microarray gene expression database of ovarian cancer subtypes (http://csiovdb.mc.ntu.edu.tw/CSIOVDB.html) is a transcriptomic microarray database containing 3431 human ovarian cancers. The database lists levels of gene expression for each subtype. CSIOVDB was used to check basal expression of NAMPT in ovarian cancer cells.

### Ovarian cancer spheroid formation

To form spheroids, OVCAR-3, SKOV-3, and KGN cells (6000, 5000, and 9000 cells per spheroid, respectively) were transferred to media supplemented with 10% FBS containing a methylcellulose solution (Sigma-Aldrich) at a final concentration of 0.25%. Next, the cells were seeded in 96-well CellStar U-bottom plates (Greiner Bio-One, Kremsmünster, Austria), centrifuged for 10 min, and incubated for 72 h to allow spheroid formation.

### Visfatin cell synthesis analysis

To examine visfatin synthesis, non-cancerous ovarian cells and ovarian cancer cells were cultured for 48 h in appropriate medium. Basal visfatin levels in cell lysates was measured after 48 h of culture using a commercial human visfatin SimpleStep ELISA Kit (ab264623, Abcam). Absorbance was measured using a ELx808 microplate reader (Bio-Tek Instruments, Winooski, VT, USA). Data were analyzed using the KC Junior software (Bio-Tek) and normalized to total protein levels in cells (measured using the Bradford method; Bio-Rad Protein, Hercules, CA, USA).

### Cell viability assay

OVCAR-3, SKOV-3, and KGN spheroids were exposed to visfatin at concentrations of 10, 50, 100, 500, or 1000 ng/mL, or to FK866 at concentrations of 10 nM or 100 nM, for 48 h, and cell viability was estimated using the CellTiter-Glo 3D cell viability assay (Promega, Charbonnières-les-Bains, France). Cells were lysed according to the manufacturer’s instructions. Luminescence was measured using a SpecttraMax L luminometer (Molecular Devices, San Jose CA, USA).

### Measurement of caspase 3/7 activity in a 3D cell culture system

The Caspase-Glo® 3/7 Assay System (Promega) was used to measure caspase 3/7 activity after treatment of OVCAR-3, SKOV-3, and KGN spheroids for 24 h with visfatin at concentrations of 10, 50, 100, 500, or 1000 ng/mL, or with FK866 at concentrations of 10 nM or 100 nM, in medium without FBS (Biowest). The Caspase-Glo® 3/7 assay provides a proluminescent caspase 3/7 DEVD-aminoluciferin substrate and a proprietary thermostable luciferase in a reagent optimized for caspase-3/7 activity. The amount of luminescence was monitored in a luminometer (SpectraMax L Luminescence Microplate Reader, Molecular Devices).

### Caspase -3, - 8 and - 9 activity assays

KGN and SKOV-3 cells were exposed to visfatin for 24 h at concentrations 10, 100, or 1000 ng/ml in medium without FBS (Biowest). The medium was removed and the cells were lysed with the caspase assay buffer. The amount of protein in the lysates was measured using the reactive compound fluorescamine (MP Biomedicals, Illkirch Cedex, France). An equal amount of cytosolic extract (50 μg of protein) from each sample was analyzed. The assay was performed by addition of 100 μM of the substrate acetylo-DEVD-amino-4-methylcoumarin (Ac-DEVDAMC; Sigma-Aldrich), which is hydrolyzed by Caspase-3, 100 μM N-Acetyl-Ile-Glu-Thr-Asp-7-Amido-4-methylcoumarin (IETD-pNA) (Sigma-Aldrich), which is hydrolyzed by Caspase-8, or 100 μM N-Acetyl-Leu-Glu-His-Asp-7-amido-4-trifluoromethylcoumarin (Ac-LEHD-AFC) (Sigma-Aldrich), which is hydrolyzed by Caspase-9, followed by incubation at 37 °C. The amount of fluorescent product was measured continuously for 120 min using a spectrofluorometer (FLx800; Bio-Tek Instruments, Winooski, VT, USA) at an excitation wavelength of 355 nm and an emission wavelength of 460 nm. Data were analyzed using KC JUNIOR software and normalized against the level of fluorescence in vehicle-treated cells.

### Real-time PCR technique

Total RNA isolation and cDNA synthesis were carried out on cells at control and/or after treatment with visfatin (100 ng/mL) for 24 h, using the TaqMan Gene Expression Cells-to-CT kit (Applied Biosystems, ThermoFisher Scientific) according to the manufacturer’s instructions. The resulting pre-amplified cDNA preparations were analyzed by real-time PCR using the StepOnePlus real-time PCR system (Applied Biosystems, ThermoFisher Scientific) and TaqMan gene expression assays in combination with TaqMan Gene Expression Master Mix containing the ROX passive reference dye (Applied Biosystems, ThermoFisher Scientific. Real-time PCR using TaqMan Gene Expression Assays (Applied Biosystems, Thermo Fisher Scientific) was performed to measure basal expression of mRNA encoding NAMPT (Hs00237184_m1) in HOSEpiC, HGrC1, OVCAR-3, SKOV-3, and KGN cells and expression of mRNA encoding caspase-3 (Hs00234387_m1), Bax (Hs00180269_m1), Bcl-2 (Hs00236329_m1), and PARP1 (Hs00242302_m1) in KGN and SKOV-3 cells at 24 h post-treatment with visfatin (100 ng/mL), as described previously [10]. Expression was normalized to that of *GAPDH* (4310884E), and relative expression was calculated using the 2^−ΔΔCt^ method [11].

### Western blot analysis

After treating with visfatin (100 ng/mL) for 48 h, the cells were lysed in lysis buffer. Proteins were separated in 4–20% Mini-Protean TGX Precast Protein Gels (Bio-Rad, Hercules, CA, USA) and transferred to Trans-Blot Turbo Mini PVDF transfer packs (Bio-Rad) using the Trans-Blot Turbo transfer system (Bio-Rad). The blots were blocked for 1 h with 0.02 M Tris-buffered saline containing 5% BSA and 0.1% Tween 20, and then incubated overnight at 4 °C with primary antibodies (Table 1). The membranes were then washed three times in TBST (Tris-buffered saline, 0.1% Tween 20) and incubated for 1 h at room temperature with horseradish peroxidase (HRP)-conjugated secondary antibodies (Table 1) as described previously [10]. β-actin was used as a loading control (Table 1). Immunopositive bands were visualized using WesternBright Sirius Western blotting HRP substrate (Advansta, Menlo Park, CA, USA). Quantification of protein bands at three independent experiments was measured by densitometry using VisionWorks LS Acquisition and Analysis software (UVP, Upland, CA, USA).

**Table 1:** Antibodies used in Western Blot reaction

Antibody	Host species	Product number	Vendor	Dilution
Cleaved caspase-3	Rabbit	#9664T	Cell Signaling Technology	1:1000
PARP	Rabbit	#9542S	Cell Signaling Technology	1:1000
BID	Rabbit	#2002S	Cell Signaling Technology	1:1000
Bcl-2	Rabbit	ab32124	Abcam	1:1000
Bax	Rabbit	ab32503	Abcam	1:1000
β-actin	Mouse	A5316	Sigma-Aldrich	1:5000
Anti-rabbit	Goat	#7074	Cell Signaling Technology	1:1000
Anti-mouse	Horse	#7076	Cell Signaling Technology	1:1000

### Quantification of mitochondrial activity

To measure the amount of ATP in OVCAR-3 and KGN cells treated with NAMPT (50 ng/mL), cells were treated with FK866 (10 nM), or co-treated with NAMPT (50 ng/mL) and FK866 (10 nM), and used in the Cell Titer-Glo Assay (Promega, Charbonnieresles-Bains, France) to assess the presence of metabolically active cells. OVCAR-3 and KGN cells were lysed according to the manufacturer’s instructions and luminescence was measured using a SpectraMax L luminometer (Molecular Devices, San Jose CA, USA). Furthermore, JC-1 staining was performed as an indicator of the electrochemical potential of the inner mitochondrial membrane. This fluorescent dye can be used to distinguish active and inactive mitochondria in the same cell. After entering the mitochondria, fluorescence changes from green (JC1 monomers, indicative of inactive mitochondria) to red as the mitochondrial membrane becomes polarized and aggregates of JC-1 form (indicative of active mitochondria). The ratio of this green/red fluorescence is independent of mitochondrial shape, density, or size, but depends only on the membrane potential (for details see Chazotte [12] and Krawczyk et al., [13]). KGN cells treated with visfatin and/or FK866 as described above were incubated with 10 μg/mL JC-1 (Sigma-Aldrich) at 37 °C in serum-free medium for 10 min. Thereafter, the medium was replaced by fresh medium and fluorescence was detected using an Axiocam 503 bright field/fluorescence microscope (Zeiss). Both monomeric (excitation wavelength, 490 nm; emission wavelength, 500–550 nm) and aggregated (excitation wavelength, 555 nm; emission wavelength, 575–620 nm) forms of JC-1 were detected. The obtained images were merged using ImageJ. A predominance of red fluorescence was indicative of active mitochondria, while a predominance of green fluorescence was indicative of inactive mitochondria.

### Statistical analysis

Statistical data analysis was performed using tools within Prism software (GraphPad Software Inc., San Diego, USA). The mean ± SEM or SD of datasets generated in triplicate (at least three independent experiments per condition) were compared using one-way or two-way analysis of variance, followed by Tukey’s test, or using a parametric Student’s *t* test (*P < *0.05*, **P < 0.01, ***P < 0.001).

## Results

### Basal level of visfatin in ovarian cancer cells from the databases

**Fig. 1:**
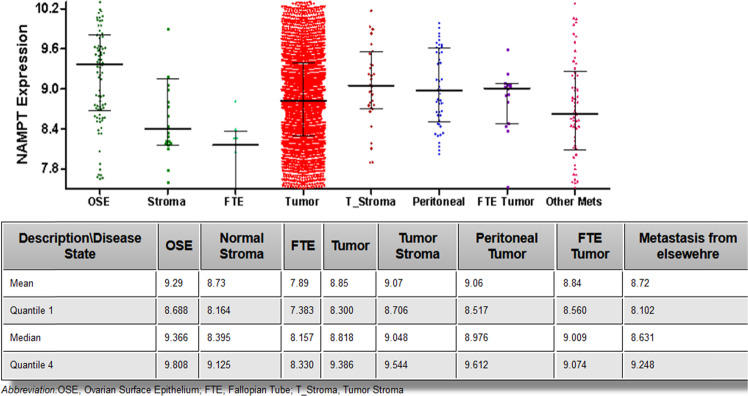
Level of visfatin in ovarian surface epithelium (OSE), normal stroma, fallopian tube epithelium (FTE), tumor, tumor stroma, peritoneal tumor, FTE tumor, and metastasis (http://csiovdb.mc.ntu.edu.tw/pages/CSIOVDB_NAMPT.html)

Visfatin expression in normal ovarian and ovarian cancer cells was checked with the help of CSIOVDB, which is a microarray gene expression database of ovarian cancer subtypes (http://csiovdb.mc.ntu.edu.tw/CSIOVDB.html) [14]. Analysis using CSIOVDB identified expression of NAMPT in each analyzed sample. The level of visfatin is significantly higher in OSE than in normal stroma. However, there were no differences in the level of NAMPT according to the type of ovarian tumor. Interestingly, that database also revealed expression of mRNA encoding NAMPT in peritoneal metastases (Fig. 1).

### Basal levels of visfatin in non-cancer and cancer ovarian cell lines

**Fig. 2:**
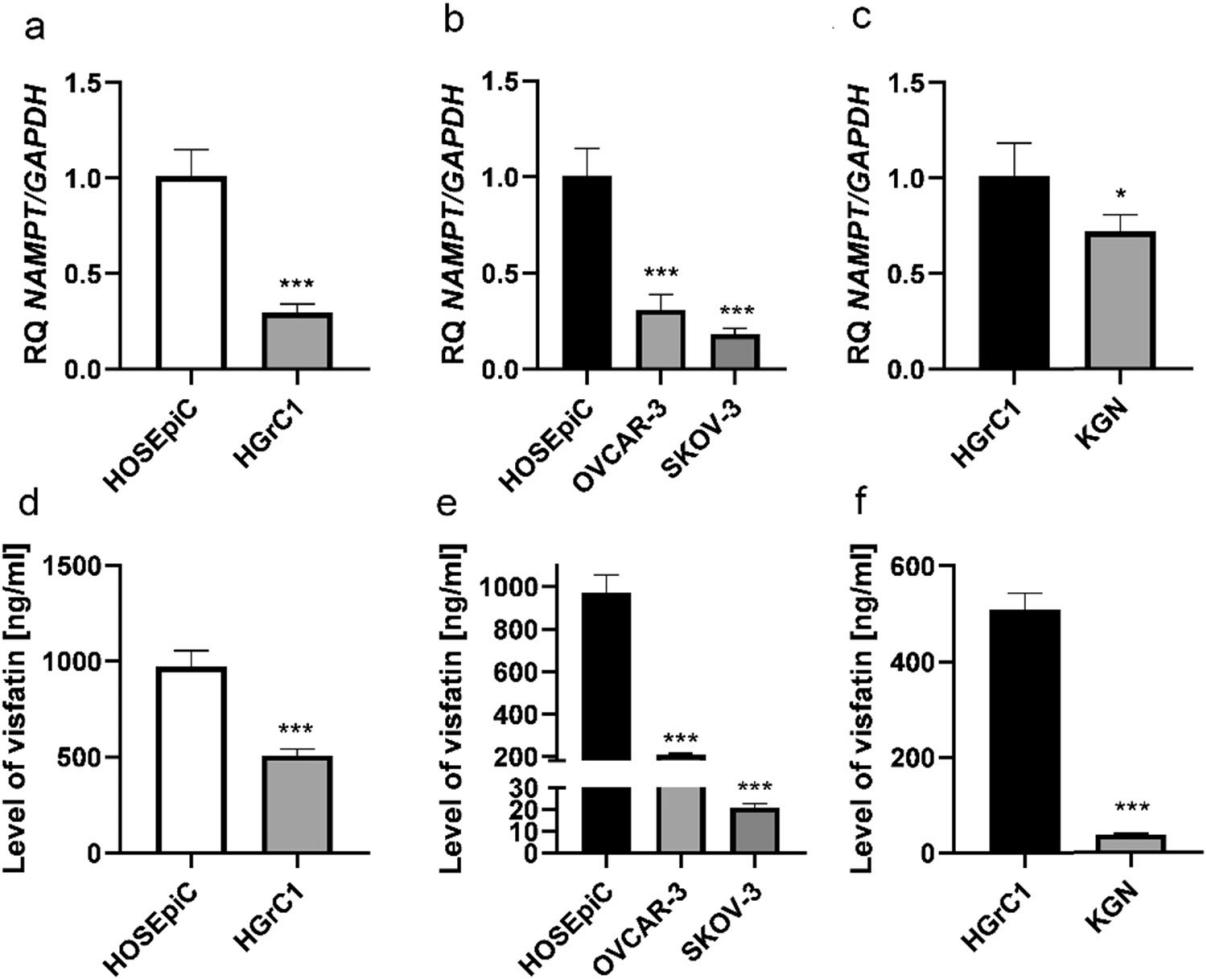
Basal levels of visfatin in non-cancer and cancer ovarian cell lines. Basal expression of mRNA encoding visfatin (NAMPT), and visfatin synthesis levels, in human ovarian non-cancer epithelial (HOSEpiC) and granulosa (HGrC1) ovarian cell lines (**a**, **d**), and in epithelial ovarian cancer cell lines (OVCAR-3) and (SKOV-3) (**b**, **e**), compared with HOSEpiC; and in ovarian granulosa tumor (KGN) cells compared with HGrC1 (**c**, **f**). Expression of NAMPT mRNA in HOSEpiC cells was set to 1.0 (RQ = relative quantity) (**a**, **b**), and expression of NAMPT mRNA in HGrC1 cells was set to RQ = 1.0. **c** Each bar represents the mean ± SEM of three independent experiments. *P < 0.05, **P < 0.01, and ***P < 0.001

Based on the data obtained from the databases, we investigated the role of visfatin in ovarian cancer tumors. To do this we measured basal expression of visfatin (NAMPT) mRNA in human ovarian non-cancer (HOSEpiC and HGrC1) and human ovarian cancer (OVCAR-3, SKOV-3, and KGN) cell lines by qRT-PCR. The relative quantity (RQ) of NAMPT mRNA in non-cancer epithelial ovarian cells (HOSEpiC) was higher than that in granulosa ovarian cells (HGrC1) by 3.45-fold (Fig. 2a; ***P < 0.001). Furthermore, the NAMPT transcript level was higher in normal epithelial ovarian cells (HOSEpiC) than epithelial ovarian cancer cell lines derived from ascites (OVCAR-3 and SKOV-3) by 3.22- and 5.56-fold, respectively (Fig. 2b; ***P < 0.001). Similarly, non-cancer ovarian granulosa cells (HGrC1) showed 1.39-fold higher expression of NAMPT mRNA than granulosa cell tumors (KGN cells) (Fig. 2c; *P < 0.05). Moreover, we examined basal synthesis of visfatin using the enzyme immunoassay Visfatin ELISA Kit. The results for the tested non-caner and cancer ovarian cell lines were similar to the mRNA expression results. Visfatin synthesis in non-cancer cells derived from human ovary HOSEpiC and HGrC1 cells (970.37 ng/mL and 508.28 ng/mL, respectively) was higher than in ovarian cancer cell lines OVCAR-3 (208.5 ng/mL), SKOV-3 (20.97 ng/mL) and KGN (37.52 ng.ml) (Fig. 2d–f; ***P < 0.001).

### Effect of visfatin on the proliferation/apoptosis ratio in ovarian cancer cell lines

**Fig. 3:**
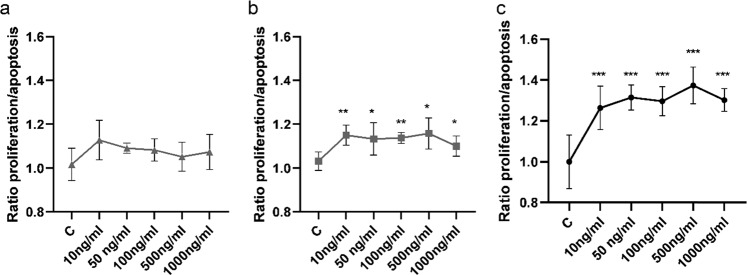
Effect of visfatin on the proliferation/apoptosis ratio in OVCAR-3, SKOV-3 and KGN cells. Dose-dependent effect of visfatin (10, 50, 100, 500, or 1000 ng/mL) on the proliferation to apoptosis ratio of OVCAR-3 (**a**), SKOV-3 (**b**), and KGN (**c**) cells. Data represent the mean ± SD. *P < 0.05, **P < 0.01, and ***P < 0.001 compared with control untreated cells

Pro-cancerogenic activity is related to resistance to anoikis, which in terns enables cancer cells to survive. Therefore, we analyzed the effect of visfatin on the proliferation/apoptosis (P/A) ratio of OVCAR-3 (Fig. 3a), SKOV-3 (Fig. 3b), and KGN (Fig. 3c) spheroids in non-adherent cell cultures. Proliferation was evaluated by counting the number of viable cells using the CellTiter-Glo 3D cell viability assay after treatment with visfatin for 48 h. Apoptosis was estimated by measuring caspase-3/7 activity after treatment with visfatin for 24 h. The P/A ratio was then calculated. Visfatin at 10, 50, 100, 500, or 1000 ng/mL increased the P/A ratio of SKOV-3 cells by 1.15-, 1.13-, 1.14-, 1.16-, and 1.10-fold, respectively, and that of KGN cells by 1.26-, 1.31-, 1.30-, 1.37-, and 1.30- fold, respectively (Fig. 3b, c; *P < 0.05, **P < 0.01, and ***P < 0.001). Visfatin had no effect on the P/A ratio of epithelial ovarian cancer (OVCAR-3) cells (Fig. 3a).

### Visfatin inhibits caspase-3 expression and activity in ovarian cancer cells

**Fig. 4:**
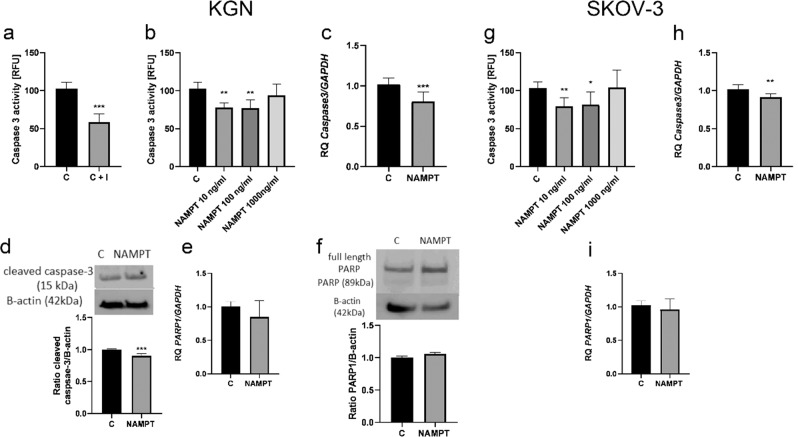
Effect of visfatin on apoptosis of KGN and SKOV-3 cells. Caspase-3 activity after treatment with a caspase-3 inhibitor (**a**). Dose-dependent effect of visfatin (10, 100 and 1000 ng/mL) on caspase-3 activity in KGN (**b**) and SKOV-3 cells (**g**). Expression level of caspase -3 (**c**, **h**) and PARP1 (**e**, **i**) mRNA in KGN and SKOV-3 cells treated with visfatin (100 ng/mL) for 24 h, and levels of cleaved caspase-3 (**d**) and PARP1 (**f**) protein in KGN cells. Expression was set to RQ = 1.0 in vehicle-treated controls. Each bar represents the mean ± SEM of three independent experiments. *P < 0.05, **P < 0.01, and ***P < 0.001

Furthermore, we examined the activity and mRNA expression of caspase-3 after visfatin treatment. As shown in Fig. 4b, visfatin at 10 and 100 ng/mL decreased caspase-3 activity from 100% to 77.8% and 76.9%, respectively, in KGN cells (**P < 0.01). Visfatin at 10 and 100 nM also decreased activity of caspase-3 from 100% to 79.2% and 81.5%, respectively, in SKOV-3 cells (Fig. 4g; *P < 0.05, **P < 0.01). The results also show that visfatin reduced expression of caspase 3 mRNA in KGN and SKOV-3 cells from 100% to 80.8% and 91.3% of that in controls, respectively (Fig. 4c, h; **P < 0.01; ***P < 0.001). Moreover, visfatin had no effect on expression of PARP1 mRNA (Fig. 4e, i), or on PARP protein cleavage (Fig. 4f).

### Visfatin inhibits apoptosis in ovarian cancer cells via the intrinsic and extrinsic pathways

**Fig. 5:**
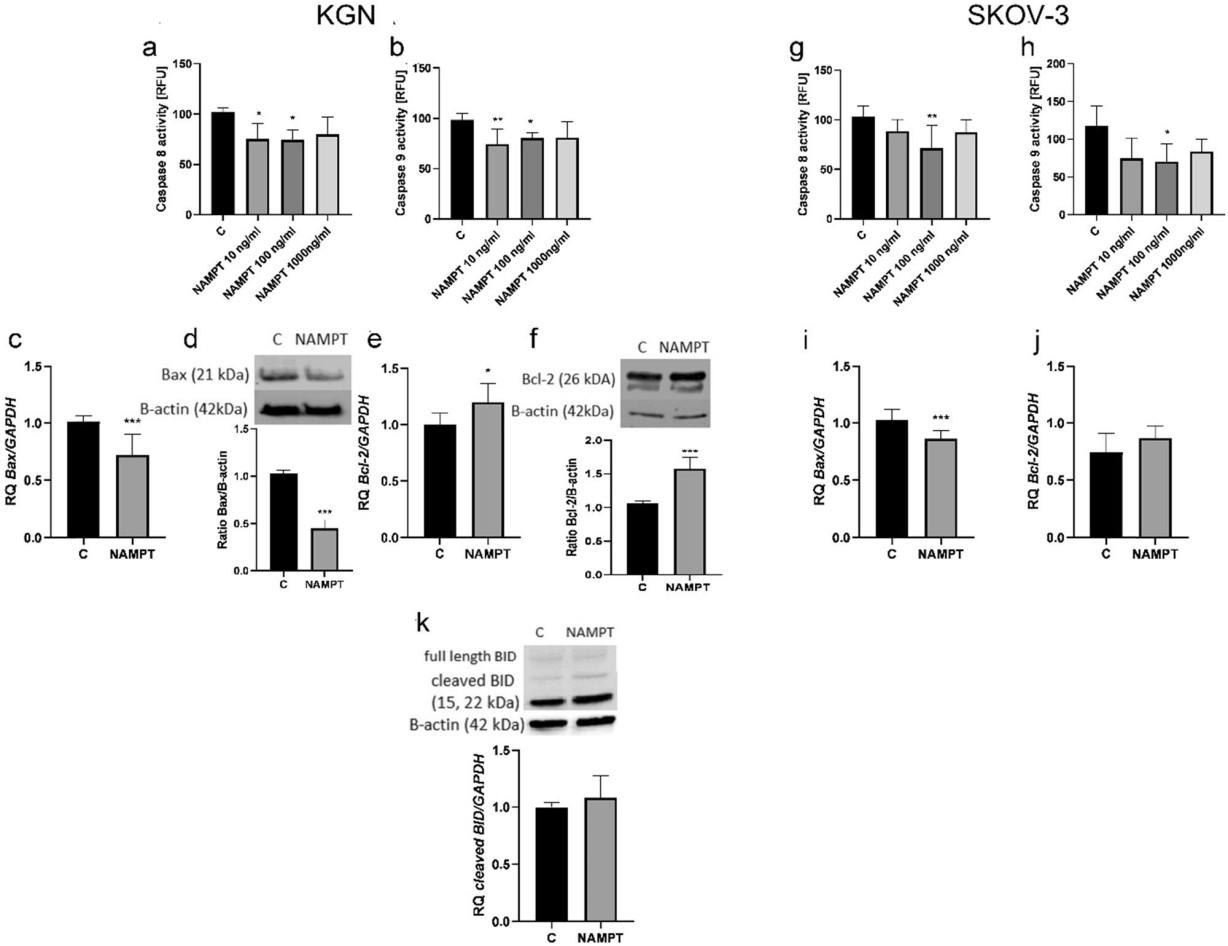
Effect of visfatin on the intrinsic and extrinsic apoptotic pathways in KGN and SKOV-3 cells. Dose-dependent effect of visfatin (10, 100, and 1000 ng/mL) on activity of caspase-8 (**a**, **g**) and caspase-9 (**b**, **h**) in KGN and SKOV-3 cells, respectively. Expression of Bax (**c**, **d**, **i**) and Bcl-2 (**e**, **f**, **j**) mRNA in KGN and SKOV-3 cells treated with visfatin (100 ng/mL) for 24 h, and levels of BID/tBID protein (**k**) in KGN cells. Expression of mRNA in vehicle-treated controls was set to RQ = 1.0. Each bar represents the mean ± SEM of three independent experiments. *P < 0.05, **P < 0.01, and ***P < 0.001

To analyze the molecular mechanism in more detail, we asked whether visfatin affects caspase-8 and -9 activity. We found that visfatin used at 10 nM and 100 nM decreased caspase-8 activity from 100% to 75.1% and 74.4%, and caspase-9 activity to 74.4% and 81.2%, in KGN cells (Fig. 5a, b *P < 0.05 and **P < 0.01). Similarly, visfatin used at 100 ng/mL reduced caspase-8 activity from 100% to 71.5% and caspase-9 activity to 70.3% in SKOV-3 cells (Fig. 5g, h; *P < 0.05 and **P < 0.01). Furthermore, we observed decreased expression of Bax mRNA and protein in KGN and SKOV-3 cells, and increased expression of Bcl-2 mRNA and protein in KGN cells (Fig. 5c–f, i–j; *P < 0.05 and ***P < 0.001). In addition, our analysis indicated activation of the BID/tBID proteins (Fig. 5k).

### FK866 reduces the proliferation/apoptosis ratio and triggers mitochondrial dysfunction, leading to decreased levels of ATP in KGN cells

**Fig. 6:**
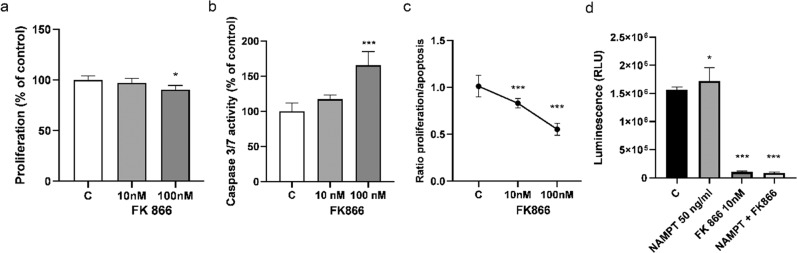
Action of visfatin inhibitor (FK866) in KGN cells. Effect of the FK866 (10 and 100 nM) on cell viability (**a**), caspase 3/7 activity (**b**), and the P/A ratio (**c**) in KGN cells. Effect of visfatin (50 ng/mL), FK866 (10 nM), and co-treatment visfatin and FK866 on the ATP content of KGN cells (**d**). Each bar represents the mean ± SEM of three independent experiments. *P < 0.05 and ***P < 0.001

To evaluate the effect of NAD + depletion on ovarian cancer survival, KGN cells were treated with FK866 (10 or 100 nM). As shown in Fig. 6a, KGN cells exposed to FK866 (100 nM) showed decreased cell viability (90% ± 4.2 living cells) compared with the control (100% ± 4.1 living cells) (Fig. 6a; *P < 0.05). Simultaneously, we observed increased (1.65-fold) caspase 3/7 activity upon treatment with FK866 (100 nM) (Fig. 6b; ***P < 0.001). Moreover, we found that FK866 at concentrations of 10 nM and 100 nM significantly reduced this P/A ratio in non-adherent cultures (by 1.2- and 1.82-fold, respectively) (Fig. 6c; ***P < 0.001). Mitochondria play key roles in apoptosis in mammalian cells; therefore, we examined the effects of visfatin and a visfatin inhibitor (FK866). ATP content was measured using the Cell Titer-Glo Assay in KGN cells. The ATP content in KGN cells treated with visfatin increased by 1.10-fold), and decreased in KGN cells by 15.79-fold, after treatment with FK866 (Fig. 6d; *P < 0.05 and ***P < 0.001). Co-treatment with FK866 and visfatin did not change ATP levels (Fig. 6d; ***P < 0.001).

### Visfatin increases mitochondrial activity, but FK866 decreases mitochondrial activity

**Fig. 7:**
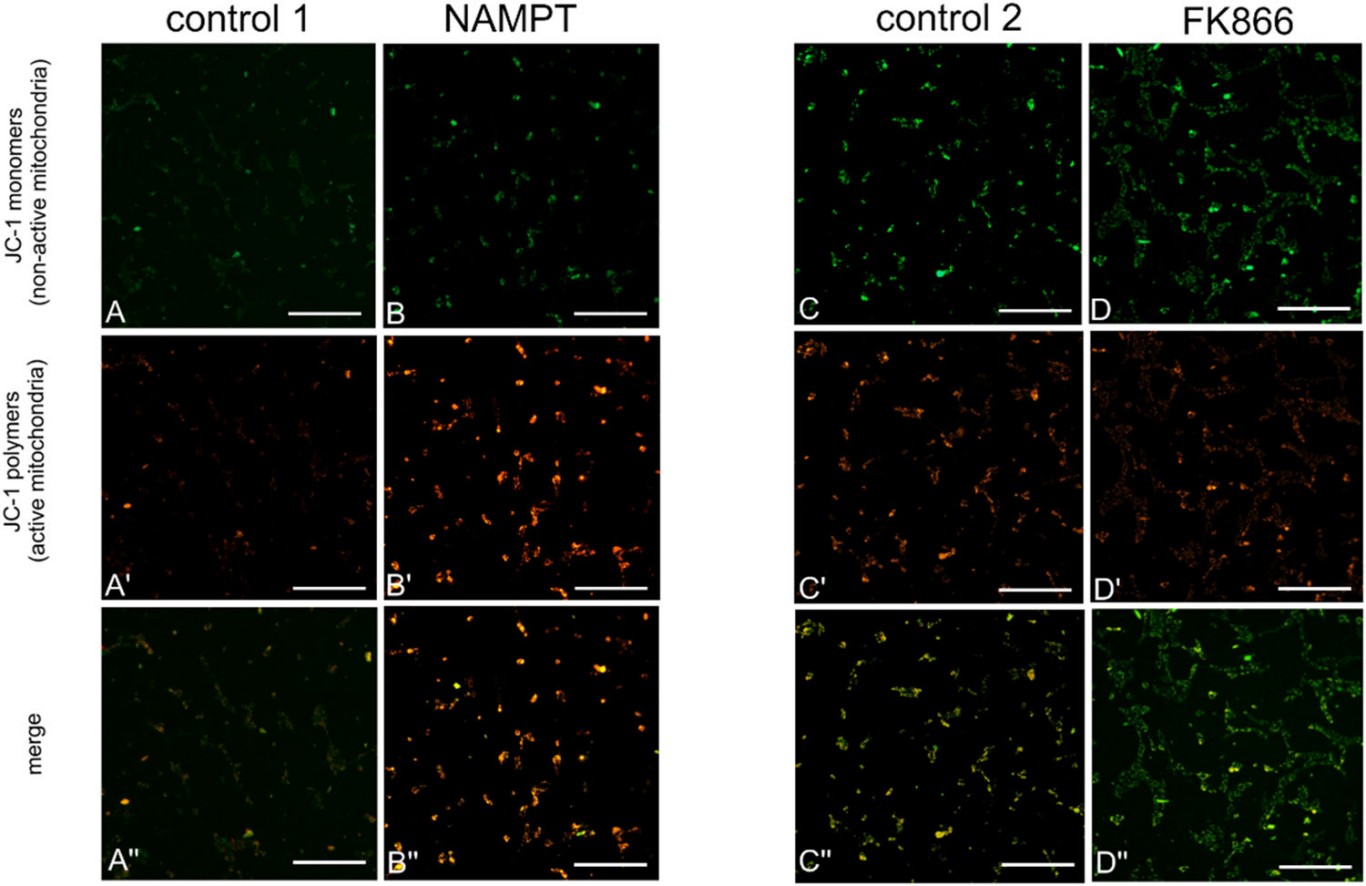
Effect of NAMPT and FK866 on mitochodrial activity in KGN cells. Mitochondrial activity in NAMPT- (**B**, **B**’, and **B**”) and FK866- (**D**, **D**’, and **D**”) treated KGN cells. Control 1 (**A**, **A**’, and **A**”) represents cells incubated with medium only, while control 2 (**C**, **C**’, and **C**”) represents cells incubated with medium plus DMSO (0.01%). Such distinctions reflect real experimental conditions. Treatment with visfatin (NAMPT) increased red fluorescence (representing active mitochondria with a high potential) (**B**’); therefore, red fluorescence is predominant in the merged image (**B**”). Treatment with the visfatin inhibitor FK866 decreased red fluorescence (representing active mitochondria) (**D**’) and increased green fluorescence emitted from inactive organelles (**D**); therefore, green fluorescence is predominant in the merged image (**D**”). Scale bar: 100 μm

Because the ATP level in KGN cells increased markedly after visfatin treatment, we decided to check whether these changes are associated with mitochondrial activity and the chemical potential of an inner membrane of these organelles. Therefore, cells pre-treated with visfatin (NAMPT) and/or its inhibitor (FK866) were stained with JC1. Use of this chemical compound enables precise distinction between active and inactive mitochondria (for further clarification see Material and Methods, and Chazotte [12]). Inactive mitochondria with a low membrane potential emitted green fluorescence (Fig. 7A–D), while active mitochondria with a high electrochemical potential emitted red fluorescence (Fig. 7A’-D’). Our analyses showed that treatment with NAMPT resulted in a significant increase in mitochondrial activity (Fig. 7B’), which can be clearly seen in merged images with predominant red fluorescence (representing active mitochondria with a high membrane potential) (Fig. 7B”). By contrast, treatment with FK866 led to a marked reduction in mitochondrial activity (Fig. 7D), resulting in predominant green fluorescence in the merged image (Fig. 7D”). It is interesting to note that when cells were treated simultaneously with both visfatin and FK866, no increase in activity was observed (not shown).

## Discussion

Obesity-related cancers, including ovarian cancer, secrete abnormal levels of adipokines such as visfatin [15, 16]. Furthermore, visfatin is present in ascites fluid from ovarian cancer patients, suggesting a connection with intraperitoneal dissemination [7]. Data obtained from databases indicate that both normal and various types of cancer ovarian cells express NAMPT. Databases show higher levels of NAMPT transcripts in OSE cells than in stroma cells, which is consistent with our results showing higher mRNA expression and secretion of visfatin by epithelial ovarian HOSEpiC cells (which are OSE cells) than ovarian granulosa HGrC1 cells (which are stroma cells). Moreover, NAMPT is expressed by cells taken from peritoneal metastases. However, no study has examined NAMPT expression in ovarian granulosa cell tumors, which are rare but can metastasize aggressively to the peritoneal cavity. Our results showed that visfatin is synthesized and secreted by ovarian cancer cell lines from ascitic fluid, and by adult granulosa cell tumor cells (KGN cells). These findings agree with reports showing that visfatin is produced by ovarian cancer cells [7]. In addition, the expression level varied between non-cancer cells and cancer cells: higher basal levels of NAMPT transcripts and higher secretion were noted in non-cancer epithelial ovarian cells (HOSEpiC) than in epithelial ovarian cancers (OVCAR-3) and (SKOV-3), and in non-cancer ovarian granulosa cells (HGrC1) than in adult granulosa (KGN) cells. The results of a previous meta-analysis revealed significantly higher circulating visfatin levels in the serum in patients with various cancers than in controls [17]. However, our results focusing on local visfatin sources indicate an inverse correlation. This suggests that local levels of visfatin may be different to circulating levels.

Ascites fluid contains single ovarian cancer cells and spheroid-like structures; the latter are thought to favor peritoneal dissemination [2]. Floating ovarian cancer spheroids acquire the ability to survive in a non-adherent state because they are resistant to anoikis. Because visfatin possesses proliferative, anti-apoptotic, pro-angiogenic, and enzymatic activity properties [18], we examined its effect on the P/A ratio in spheroids of three ovarian cancer cell lines grown under non-adhesion conditions to reflect in vivo conditions. We observed that visfatin increased the P/A ratio in SKOV-3 and KGN cells. However, there was no effect in the OVCAR-3 cell line, which expressed the highest levels of visfatin. After observing the anti-apoptotic effects of the visfatin, we evaluated its effect on caspase activation. We found downregulated expression of caspase-3 mRNA, accompanied by decreased caspase-3 activity, in KGN and SKOV-3 cells. In addition, we showed decreasing cleavage of caspase-3 following visfatin treatment. Wang et al. [8] reported that visfatin stimulates proliferation, and inhibits apoptosis of, endometrial carcinoma in both Ishikawa and KLE cells. Furthermore, visfatin may promote proliferation and inhibit apoptosis of colon and breast cancer cells [9, 19]. PARP is one of the best known substrates for caspase activity, and PARP cleavage to yield fragments of 89 and 24 kDa is a universally accepted hallmark of apoptosis [20]. Our analysis of PARP1 expression in KGN and SKOV-3 cells showed no changes in NAMPT gene expression, or the cleaved form PARP1 protein, after visfatin treatment. These observations are partly in line with those of Gholinejad et al. [9], who reported that visfatin improves cell viability, and prevents TNF-α-induced apoptosis and PARP cleavage, in breast cancer cells.

Induction of anoikis occurs through interplay between two apoptosis pathways: the intrinsic pathway and the extrinsic pathway. The extrinsic pathway is activated by upregulated FAS and FasL (and inhibited by FLIP), leading to activation of caspase-8, followed by activation of caspase-7 and caspase-3. Loss of cell adhesion also activates the intrinsic pathway by increasing activation of pro-apoptotic proteins such as Bax, which inactivate anti-apoptotic Bcl-2 proteins and release cytochrome c from the mitochondria, which activates caspase 9, followed by caspase 3 [21]. Our results showed that visfatin reduces both caspase 8 and caspase 9 activity in KGN and SKOV-3 cells. Additionally, we found downregulated Bax expression and upregulated Bcl-2 expression at the gene and protein levels. This consistent with experiments showing that visfatin decreases expression of pro-apoptotic proteins (Bax and cleaved caspase) and simultaneously increases expression of anti-apoptotic proteins (Bcl-2), culminating in neuroprotective effects against ischemic injury [22]. Similarly, Xiang et al. [23] showed that visfatin protects rat pancreatic β-cells against IFN-γ-induced apoptosis. The link between the two apoptosis pathways is the BID protein, which is activated after cleavage of caspase-8 [24, 25]. Interestingly, we noted increased expression of cleaved BID, suggesting that both the intrinsic and extrinsic pathways are involved in the action of visfatin. Thus, visfatin plays a role in anoikis resistance by inhibiting both the intrinsic and extrinsic pathways.

To confirm our observations, we used the nicotinamide analog FK866 (also known as WK175 and APO866) which acts as a competitive inhibitor of NAMPT [26]. FK866 was the first nanomolar-effective NAMPT inhibitor, and is currently the most widely used in the clinic. FK866 has shown positive results when used to treat cancer [27]. We found that FK866 (100 nM) significantly inhibited proliferation, and increased apoptosis, of KGN cells. Moreover, the P/A ratio decreased after treatment with FK866. In vitro studies show that FK866 induces apoptosis of liver cancer cells (HepG2) via highly specific, non-competitive inhibition of nicotinamide phosphoribosyltransferase (NAPRT) [28]. Furthermore, FK866 inhibits growth of a wide variety of cancer cell lines, and of tumors in in vivo models [29, 30].

The role of mitochondria in caspase activation during apoptosis is fairly well characterized. However, disruption of mitochondrial function during apoptosis mediated by caspase cleavage has also been demonstrated [31]. Waterhouse et al. [32] reported mitochondrial damage and a rapid fall in ATP levels in apoptotic cells upon caspase activation. Therefore, we next analyzed the effect of NAMPT and FK866 on mitochondrial function. We observed increased the ATP content after treatment of KGN cells with visfatin, whereas FK866 decreased ATP levels. Gherke et al. [33] reported that FK866 triggers death of chronic lymphocytic leukemia cells by reducing cellular NAD and ATP levels in a time- and concentration-dependent manner. Therefore, to detect changes in mitochondrial activity, we performed JC-1 staining. Our experiments showed that visfatin increased, and FK866 decreased, mitochondrial activity. These results are in line with a previous observation that FK866 inhibits mitochondrial respiratory activity [28]. Interestingly, a previous study showed that a NAMPT inhibitor induced apoptosis accompanied by activation of caspases, DNA fragmentation, and disruption of mitochondrial transmembrane potential, in primary adult T-cell leukemia/lymphoma (which shows high expression of NAMPT) [34]. Consistent with this, Liu et al., [30] demonstrated that targeting of NAD + by FK866 reduced mitochondrial membrane potential, which ultimately increased apoptosis, and inhibited proliferation, of gastric cancer cells. In addition, NAMPT silencing reduced intracellular NAD and ATP levels [30].

To sum up, the observations that ATP levels and mitochondrial activity increase in parallel with downregulation of caspase expression after treatment with visfatin support the hypothesis that visfatin is an ani-apoptotic factor that triggers anoikis resistance in ovarian cancer spheroids. The visfatin inhibitor FK866 exerted pro-apoptotic properties by decreasing ATP levels and mitochondrial activity in parallel with upregulating caspase activity, thereby stimulating anoikis in ovarian cancer spheroids. Thus, targeting/inhibiting visfatin may be a potential novel therapeutic approach to inhibiting peritoneal dissemination of ovarian carcinoma.
